# Evaluation of Trust Within a Community After Survivor Relocation Following the Great East Japan Earthquake and Tsunami

**DOI:** 10.1001/jamanetworkopen.2020.21166

**Published:** 2020-11-05

**Authors:** Krisztina Gero, Jun Aida, Katsunori Kondo, Ichiro Kawachi

**Affiliations:** 1 Department of Social and Behavioral Sciences, Harvard T. H. Chan School of Public Health, Boston, Massachusetts; 2 Department of Health Sciences, Bouvé College of Health Sciences, Northeastern University, Boston, Massachusetts; 3 Department of International and Community Oral Health, Tohoku University Graduate School of Dentistry, Miyagi, Japan; 4 Center for Preventive Medical Sciences, Chiba University, Chiba, Japan; 5 Center for Gerontology and Social Science, National Center for Geriatrics and Gerontology, Aichi, Japan

## Abstract

**Importance:**

Trust is a core component of social cohesion, facilitating cooperation and collective action in the face of adversity and enabling survivors to remain resilient. Residential stability is an important prerequisite of developing trusting relations among community members. However, little is known about whether the movement of internally displaced persons (IDPs) after a disaster might change community relations.

**Objective:**

We explored perceived changes in trust within 1 community directly impacted by the 2011 Great East Japan Earthquake and Tsunami.

**Design, Setting, and Participants:**

This prospective cohort study examined survey data from 3594 residents of Iwanuma City, Japan, aged 65 years or older. Data were obtained from the Iwanuma Study—part of the Japan Gerontological Evaluation Study, a nationwide cohort study established in 2010—approximately 7 months before the disaster. All Iwanuma City residents age 65 years or older (8576 residents) were eligible to participate in 2010. The response rate was 59.0% (5058 residents). A follow-up survey was conducted in 2013, approximately 2.5 years after the disaster. Of the 4380 remaining participants who answered the baseline survey, 3594 were recontacted (follow-up rate, 82.1%). Data analysis was performed from July 1, 2019, to January 9, 2020.

**Exposures:**

The number of temporarily relocated Iwanuma City survivors within 100 m and 250 m of a nonrelocated resident’s home address.

**Main Outcomes and Measures:**

Perceived changes in particularized trust (ie, trusting people from the same community) and generalized trust (trusting people from other communities) measured on a 5-point Likert scale.

**Results:**

Among 3250 nonrelocated residents (1808 [55.6%] women; mean [SD] age, 76.5 [6.2] years) of Iwanuma City included in the analytic sample, multivariable-adjusted multinomial logistic regression analyses found that each standard deviation increase in the influx of internally displaced persons (1 SD = 11 IDPs) within 250 m of a resident’s home address was associated with higher odds of a decrease in the resident’s particularized and generalized trust (odds ratio, 1.17; 95% CI, 1.04-1.32).

**Conclusions and Relevance:**

The influx of IDPs in the host community appeared to be associated with an erosion of trust among locals. To avoid the erosion of social cohesion after a disaster, it may be crucial to provide opportunities for social interaction between old and new residents of communities.

## Key Points

**Question:**

How is the movement of internally displaced survivors in the aftermath of a disaster associated with perceived trust towards others within a host community?

**Findings:**

In this cohort study that included 3250 adults aged 65 years or older, each standard deviation increase in the influx of temporarily relocated survivors within 100 m of a resident’s home address was associated with a decrease in their trust in both people from their community and outside of it.

**Meaning:**

The findings of this study suggest that opportunities for social interaction between old and new residents of host communities may be crucial for maintaining social trust.

## Teaser

This cohort study examined levels of community trust in survey responses from communities in Iwanuma City, Japan, hosting resettled survivors of the 2011 Great East Japan Earthquake and Tsunami.

## Introduction

The long-term health sequelae of disasters range from mental health problems (eg, depression, anxiety, posttraumatic stress disorder, psychological distress) to functional decline (eg, cognitive and/or physical disability).^1,2,3,4,5^ A critical feature of communities that enable survivors to remain resilient in the face of adversity is the strength of social connections, known as social capital.^4,6,7,8^ Tightly knit communities are more effective in mobilizing assistance to the neediest members of the group, before even first responders have reached the scene, and long before formal mechanisms of insurance can become activated. During the recovery phase of disasters, socially cohesive groups are more effective in giving voice to the needs of the community, and in preventing the exit of members from disaster-stricken areas.^9^

Unfortunately, disasters are also commonly associated with major disruptions in social connections through the residential displacement of survivors. Such disruption of social relations affects not only the survivors who become separated from their predisaster communities, but also the host communities, which have to deal with a large influx of internally displaced persons (IDPs). Previous studies have documented the effects of residential dislocation on the social connections of disaster victims.^10^ However, few studies have focused on the impacts on communities at the receiving end of large inflows of IDPs. In the 2011 Great East Japan Earthquake and Tsunami, nearly a quarter of a million residents were forced to relocate to new communities. Japanese media at the time highlighted instances of local friction caused by the large internal flows of disaster victims into nearby communities.^11,12^ However, to our knowledge, no systematic attempt has been made to document the impact of mass residential relocation on the quality of social connections in the host community.

In this study, we took advantage of a unique natural experiment stemming from the 2011 disaster. The city of Iwanuma (population 44 000) in Miyagi prefecture, located 80 km to the west of the earthquake epicenter, was one of the field sites for a nationwide cohort study of healthy aging, called Japan Gerontological Evaluation Study (JAGES). Our study specifically focused on the association of disaster with the erosion of social trust. Generalized trust—the belief that most people, including strangers, can be trusted—represents a crucial component of social capital, facilitating cooperation and collective action for mutual benefit.^13^ In turn, generalized trust has been linked to better health outcomes not only in the context of disasters, but in the general population as well.^4,14,15,16,17,18,19^

Previous studies have demonstrated that generalized trust in the community is associated with factors such as residential stability and ethnic homogeneity.^20^ Residential stability is recognized as an important prerequisite for developing community trust. Trust develops over time as a result of repeated social interactions (eg, the trading of mutual favors) between members of stable communities. For example, research suggests that Japanese communities reporting the highest levels of generalized trust among residents tend to be the oldest communities in continuous existence based on historical maps.^21^ Residential homogeneity (eg, by ethnicity) is also correlated with levels of trust, especially in the United States, where studies consistently reported a negative association between ethnic diversity and social cohesion.^20^

Japan is a notably homogeneous society (by ethnicity) owing to over 2 centuries of exclusion of people born outside of Japan (beginning with the *sakoku* edict of the Tokugawa era). Nevertheless, the 2011 disaster provides a pertinent natural experiment for examining outcomes associated with the mass influx of so-called outsiders into existing communities.

In the literature, a critical distinction is drawn between particularized trust (the belief that certain known others can be trusted) vs generalized trust, which includes trust of unknown groups and strangers.^20^ We hypothesized that the influx of IDPs into a community would be associated with a decrease in generalized trust but not particularized trust. However, previous studies suggest that community heterogeneity has broader spillover effects in eroding generalized trust.^20^ Therefore, to address knowledge gaps, we explore potential differences between the associations of disaster with generalized vs particularized trust within the host community.

## Methods

### Study Population

Data were obtained from the Iwanuma Study, a part of the JAGES nationwide cohort study established in 2010 to examine factors associated with healthy aging of community-dwelling residents in 31 municipalities throughout Japan. On March 11, 2011, a magnitude 9 earthquake occurred at the Pacific coast of Tohoku prefecture, affecting one of the participating municipalities, Iwanuma City, which was located approximately 80 km west of the epicenter and in the direct line of the resulting tsunami. The disaster claimed the lives of 180 residents, flooded 48% of the town’s land area, and damaged 5542 housing units.^22^

In August 2010, around 7 months before the disaster, a census was conducted for every Iwanuma City resident aged 65 years or older identified through the local official residential registry. In October 2013, approximately 2.5 years after the disaster, the town’s residents were recontacted, and an in-home survey was conducted to gather information on the health status and social capital of the survivors.

Informed consent was obtained from participants at the time of data collection. This study excluded respondents who incompletely signed consent forms, and the analytic sample was restricted to the 3250 individuals (1442 men and 1808 women) who were not relocated due to the earthquake (eFigure in the Supplement). The study protocol was approved by the ethics committees of Nihon Fukushi University, Chiba University, Tohoku University, and the Harvard T. H. Chan School of Public Health. This study followed the Strengthening the Reporting of Observational Studies in Epidemiology (STROBE) reporting guideline.

### Social Trust

To measure perceived changes in trust in others in the wake of the earthquake, in the JAGES 2013 survey study participants from Iwanuma City were asked the following questions: (1) ”How did your trust in *people in your community* change from before the earthquake?” and (2) “How did your trust in *people from other communities* change from before the earthquake?” Of 3250 nonrelocated participants, 3216 (1787 [55.6%] women) answered the first, and 3210 (1785 [55.6%] women) answered the second question. Possible answers included “became much stronger,” “became stronger,” “did not change,” “became weaker,” and “became much weaker.”

### Number of IDPs in the Host Community

The 2013 survey also asked the following question: “Did you move to a new residence because of the earthquake?” The possible answers were: (1) “No,” (2) “The entire district was relocated to temporary housing” (*kasetsu jutaku*: similar to FEMA-style trailer homes in the US), (3) “Moved to temporary housing, but not by entire district relocation,” (4) “Moved to *minashi kasetsu* temporary housing” (rental housing on the open market), and (5) “Purchased a new home.” Because of the disaster, 131 study participants were forced to move to temporary housing, either alone (47 participants) or together with others from their district (84 participants).

Using data from the local residential registry, home address coordinates of everyone in Iwanuma city aged 65 years or older (including those who did not respond to the JAGES surveys) were verified both in 2010 and 2013, enabling us to identify a further 303 relocated residents. Using these home address coordinates for 2013, the geodetic distance between each nonrelocated and relocated participant was calculated, from which the number of IDPs within a 100 m and 250 m buffer zone of each nonrelocated resident was determined.

### Covariates

As potential confounders associated with both trust and housing location, the following baseline variables measured in 2010 were included in the multivariate-adjusted models: education (<10 or ≥10 years), employment status (paid job, retired, or never been employed), equivalized income (<¥1.49 million or ≥¥1.49 million per year [approximately US$ 14 900]), and marital status (married, divorced, widowed, or single/other). We also adjusted for variables possibly associated with perceptions of trust, including age (measured as a continuous variable in years), sex, living alone, losing a close relative or friend due to the earthquake, and depressive symptoms, all of which were measured in 2013. Equivalized income was calculated by dividing the gross annual household income by the square root of the number of household members. Depressive symptoms were measured using the short version of the Japanese Geriatric Depression Scale (GDS-15), a self-administered questionnaire consisting of 15 questions with yes or no as possible answers. With higher scores indicating higher depressive symptomatology, a GDS-15 score of 5 or more was used as a predefined cut-off point to create a binary variable.^23,24,25^

### Statistical Analysis

To assess whether IDPs were relocated to areas where residents already had low trust predisaster, levels of particularized trust (ie, measuring agreement with the question, “Do you think people living in your area can be trusted in general?” on a Likert scale ranging from 1 to 5) and generalized trust (ie, yes, no, or maybe responses to the question, “Do you feel that people can generally be trusted?”) among all participants in Iwanuma city were confirmed using the JAGES 2010 survey. Sequential regression multiple imputation (or imputation by fully conditional specification or by using chained equations) was conducted, including all variables of the final analytical model to account for missing covariate data (missingness, 0%-12.3%). After creating 10 imputed data sets, multinomial logistic regression models were used to estimate multivariate-adjusted odds ratios (OR) with 95% CIs for perceived change in trust after the earthquake. For better comparability and reliability, exposure variables were standardized. Considering that outlier values might skew the probability distribution, robust standardization (value = [value − median]/[75th percentile − 25th percentile]) was used. After confirming that the interaction terms between the explanatory variables and sex were not statistically significant, data from men and women were combined in all analyses.

Two sets of models were constructed to estimate the association between the number of IDPs in the proximity of nonrelocated residents within 100 and 250 m and perceived change in trust in others. Model 1 is a univariate model. Model 2 controlled for equivalent income, education, marital status, employment status, single residence, the death of a relative as a result of the disaster, the death of a friend as a result of the disaster, and depressive symptoms. SAS version 9.4 (SAS Institute Inc) was used to conduct all analyses. The data were analyzed from July 1, 2019, to January 9, 2020. All tests were 2-tailed, with a significance level of *P* < .05.

## Results

Of 8576 identified residents contacted to participate in 2010 who were aged 65 years or older, 5058 (59.0%) responded. Of 4380 eligible participants who answered the baseline survey, 3594 were recontacted in 2013, constituting an 82.1% follow-up rate (eFigure in the Supplement).

Table 1 presents the descriptive characteristics of Iwanuma study respondents. Among the 3250 nonrelocated participants, the mean (SD) age was 76.5 (6.2) years, and 1808 (55.6%) were women. At baseline, 1087 participants (33.5%) had less education (<10 years of educational attainment), and 997 (30.7%) reported low household income (annual income <¥1.49 million). As for disaster experiences, 808 participants (24.9%) and 464 (14.3%) lost a close relative or friend, respectively. Approximately one-third (32.6%) also reported depressive symptoms in the follow-up survey, while 70 (2.2%) had lower trust in people from other areas and 73 (2.3%) had lower trust of people in their own area. Compared with nonrelocated participants, IDPs were more likely to have low education levels (63.4% [83 participants] vs 33.5%) and low household income (51.9% [68 participants] vs 30.7%) predisaster. They were also more likely to experience the loss of a relative (56.5% [74 participants] vs 24.9%) or friend (32.8% [43 participants] vs 14.3%), and to report depressive symptoms (61.1% [80 participants] vs 32.6%) and lower trust in people from their own (15.3% [20 participants] vs 2.3%) or other communities (10.7% [14 participants] vs 2.2%) after the earthquake. There were no significant differences between all Iwanuma study respondents and the nonrelocated participants in the examined factors, except for a slightly lower proportion of nonrelocated residents with low educational attainment (all respondents, 35.8% vs nonrelocated respondents, 33.5%; *P* = .046).

**Table 1.** Participant Characteristics

Characteristic	Participants, No. (%)		
	All (n = 3567)	Nonrelocated (n = 3250)	Internally displaced (n = 131)
Women	2015 (56.5)	1808 (55.6)	80 (61.1)
2010 survey			
Low educational attainment (<10 y)	1277 (35.8)	1087 (33.5)	83 (63.4)
Retired	2228 (62.5)	2060 (63.4)	72 (55.0)
Equivalized household income <¥1.49 million/y^a^	1152 (32.3)	997 (30.7)	68 (51.9)
Married	2517 (70.6)	2310 (71.1)	91 (69.5)
Divorced	94 (2.6)	87 (2.7)	3 (2.3)
Widowed	868 (24.3)	776 (23.9)	32 (24.4)
Single or other	88 (2.5)	77 (2.4)	5 (3.8)
2013 survey			
Age, mean (SD), y	76.5 (6.2)	76.5 (6.2)	76.9 (6.6)
Living alone	407 (11.4)	374 (11.5)	17 (13.0)
Lost a close relative	936 (26.2)	808 (24.9)	74 (56.5)
Lost a close friend	549 (15.4)	464 (14.3)	43 (32.8)
GDS-15 score ≥5	1218 (34.1)	1059 (32.6)	80 (61.1)
Low trust in people from other areas	92 (2.6)	70 (2.2)	14 (10.7)
Low trust in people within the area	103 (2.9)	73 (2.3)	20 (15.3)

Abbreviation: GDS-15, Japanese Geriatric Depression Scale.

^a^ Approximately US$ 14 900.

**Figure 1.**
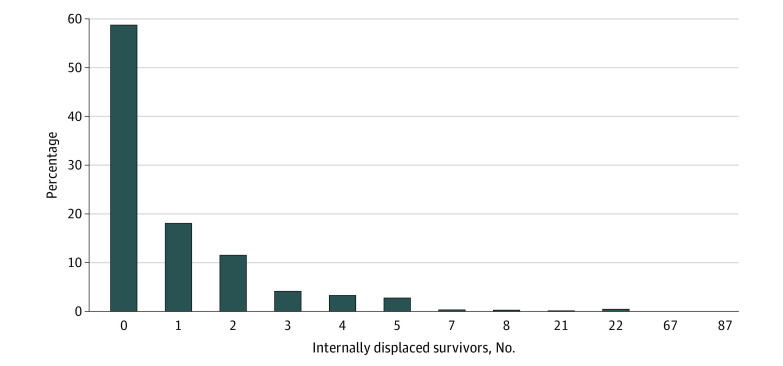
Distribution of Internally Displaced Persons Within a 100 m Buffer Zone in 2013 in Iwanuma

**Figure 2.**
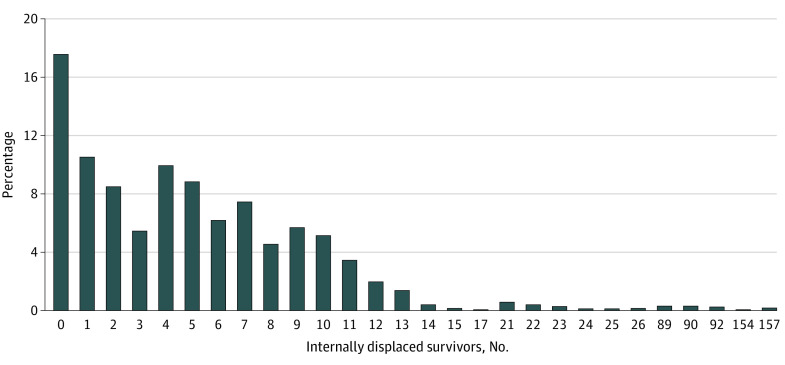
Distribution of Internally Displaced Persons Within a 250 m Buffer Zone in 2013 in Iwanuma

Figure 1 and Figure 2 display the distribution of IDPs within a 100 m and a 250 m buffer zone, respectively. The number of IDPs generally ranged from 0 to 8 within a 100 m area, and 0 to 26 within a 250 m area of the nonrelocated residents. However, owing to group relocation, some districts experienced an increased influx of displaced survivors, ranging from 21 to 87 people within a 100 m area, and 89 to 157 individuals within a 250 m buffer zone.

Table 2 shows levels of household income and generalized and particularized trust among JAGES participants in Iwanuma City before the 2011 Earthquake and Tsunami. Participants living in school districts with temporary housings built postdisaster were less likely to report low generalized trust in 2010 compared with those from other school districts. There were no significant differences in predisaster mean particularized trust scores between residents of group relocation affected and other areas. Residents from flooded communities reported a significantly lower predisaster mean household income compared with those from communities without postdisaster temporary housing units. However, there were no significant mean household income differences between the host and the disaster-effected school districts.

**Table 2.** Household Income and Levels of Generalized and Particularized Trust Predisaster Among JAGES 2010 Panel Participants

	Residents, No.			
	Communities without temporary housing units (n = 3158)	All host communities (n = 409)	Main host community (n = 133)^a^	Flooded communities (n = 565)
Particularized trust responses	3094	393	128	547
Mean (SD) Likert score, 1-5	3.8 (0.8)	3.7 (0.8)	3.6 (0.9)	3.7 (0.8)
Generalized trust responses^b^	831	49	0	59
Low trust, No. (%)	76 (9.1)	3 (6.1)	NR	6 (10.2)
Equivalized household income, ¥10 000^c^	2535	318	100	433
Continuous, mean (SD)	235.2 (152.5)	222.1 (164.5)	214.9 (181.2)	201.9 (144.8)

Abbreviations: JAGES, Japan Gerontological Evaluation Study; NR, none reported.

^a^ The school district with the highest number of internally displaced persons.

^b^ An extended version of the JAGES 2010 survey containing a question related to generalized trust was distributed to a randomly selected 20% of the study participants.

^c^ Each unit represents ¥10 000 (approximately US$ 100).

Table 3 provides multivariate-adjusted ORs with 95% CIs for change in trust after the earthquake associated with the number of IDPs in the participants’ proximity. An increase in the number of IDPs within 100 or 250 m of participants’ home addresses was associated with higher odds of a decrease in the trust of nonrelocated residents, both for people from their own and other communities. After controlling for all covariates (Model 2), the OR for “much weaker trust” after the earthquake (compared with “no change in trust”) per each standard deviation increase in the number of relocated people within 100 m (SD = 1 IDP) was 1.08 (95% CI, 1.03-1.13) related to generalized trust, and 1.08 (95% CI, 1.04-1.14) related to localized trust. The corresponding ORs with each SD increase within a 250 m radius (SD = 11 IDPs) were 1.17 (95% CI, 1.04-1.32) for both generalized and local trust. The OR estimates did not significantly change when covariates were excluded from the model (Model 1).

**Table 3.** Multivariate Adjusted ORs for Change in Trust After the Earthquake Associated With the Number of IDPs Within a 100- to 250-m Area

Responses	Trust in people from other areas			Trust in people from your area		
	Respondents, No.	IDPs Within 100 m, OR (95% CI)/1 SD (1 IDP)^a^	IDPs Within 250 m, OR (95% CI)/1 SD (11 IDPs)^a^	Respondents, No.	IDPs Within 100 m, OR (95% CI)/1 SD (1 IDP)^a^	IDPs Within 250 m, OR (95% CI)/1 SD (11 IDPs)^a^
**Model 1**^**b**^						
No change	2706	1 [Reference]	1 [Reference]	2554	1 [Reference]	1 [Reference]
Much weaker since the earthquake	12	1.07 (1.03-1.11)^c^	1.15 (1.03-1.29)^d^	12	1.07 (1.03-1.11)^c^	1.16 (1.04-1.29)^c^
Weaker since the earthquake	58	0.89 (0.73-1.09)	0.92 (0.71-1.19)	61	0.89 (0.73-1.08)	0.97 (0.80-1.17)
Stronger since the earthquake	400	0.92 (0.86-0.99)^d^	0.93 (0.85-1.02)	553	0.96 (0.91-1.01)	0.95 (0.89-1.02)
Much stronger since the earthquake	34	0.80 (0.58-1.10)	0.81 (0.49-1.34)	36	0.84 (0.62-1.12)	0.90 (0.63-1.29)
**Model 2**^e^						
No change	2706	1 [Reference]	1 [Reference]	2554	1 [Reference]	1 [Reference]
Much weaker since the earthquake	12	1.08 (1.03-1.13)^f^	1.17 (1.04-1.32)^d^	12	1.08 (1.04-1.14)^f^	1.17 (1.04-1.32)^c^
Weaker since the earthquake	58	0.89 (0.73-1.08)	0.94 (0.74-1.20)	61	0.89 (0.74-1.08)	0.98 (0.82-1.17)
Stronger since the earthquake	400	0.92 (0.86-0.99)^d^	0.94 (0.86-1.03)	553	0.96 (0.91-1.01)	0.95 (0.88-1.02)
Much stronger since the earthquake	34	0.81 (0.59-1.12)	0.84 (0.53-1.34)	36	0.83 (0.62-1.11)	0.89 (0.60-1.31)

Abbreviations: IDP, internally displaced person; OR, odds ratio.

^a^ Standard deviation calculated by robust standardization (value = [value − median]/[75th percentile − 25th percentile]) to adjust for potential outliers.

^b^ Model 1 is a univariate model.

^c^ *P* < .01.

^d^ *P* < .05.

^e^ Model 2 adjusted for age, sex, equivalized household income, education, marital status, employment status, living alone, losing a family member, losing a close friend, and depressive symptoms.

^f^ *P* < .001.

## Discussion

Our study found that after the 2011 Tohoku Earthquake, the influx of IDPs to another community was associated with weakening of both generalized and local trust, suggesting that the concentration of IDPs within a temporary shelter village (as happened in Iwanuma) may have a particularly detrimental effect on social cohesion.

Building trust between residents of a community depends on repeated social interactions over an extended period of time, whereas exposure to outsiders or out-groups can trigger conflict and mistrust.^20^ In a 2007 study, Putnam^26^ found that the influx of immigrants in communities can spur perceived competition over scarce resources (eg, housing, schools), ultimately resulting in reduced community cooperation and altruism, as well as lower trust not only in people perceived as different, but also in those who are perceived as similar. This study found that internal forced migration after a disaster, even within the same city from 1 district to another, might also lead to the erosion of the trust of nonrelocated residents in people from other communities as well as in people from the same community.

Previously, we reported that relocating IDPs together as a group, as opposed to randomly housing them throughout the community, can be an effective means of preserving social connections and strengthening the resilience of disaster survivors.^10^ However, the same policy may also inadvertently promote erosion of trust between older residents of the host community and newcomers.

We have therefore identified a potential dilemma in postdisaster resettlement. Our previous studies^10,27,28^ have reported that the resettlement of survivors needs to take into account the preexisting social ties within a disaster-effected community in order to prevent the loss of communality associated with widespread housing destruction. In Iwanuma, the city offered 2 different means of relocation to temporary housing to survivors. People could choose between individual relocation—moving to public housing by a random lottery or seeking housing in the open rental market—or group relocation, in which whole communities would be moved together as a group into prefabricated temporary housing villages (resembling FEMA-style trailer parks in the US). Families who wanted to escape the emergency shelters as soon as possible selected the individual option, so they could leave the shelters as soon as their number came up on the lottery. However, this mode of resettlement had the unintended consequence of disrupting existing social connections in the community and scattering the residents randomly throughout the trailer settlement. We previously found that people selecting the lottery option reported lower levels of social participation and social support.^10,28^ By contrast, people selecting group resettlement were even more likely to be engaged in informal social participation 3 years after the disaster compared with before the disaster.^10^ However, as the result of our present analysis suggests, the option of moving large numbers of IDPs together and concentrating them into 1 location may lead to greater friction with established residents of host communities.

### Limitations

Several limitations need to be considered while interpreting the findings of this study. First, although we controlled for socioeconomic status, depressive symptoms, and personal disaster experiences, there may be residual confounders that we failed to take into account. Second, the number of nonrelocated participants reporting much weaker trust after the earthquake is quite small (12 participants), resulting in relatively wide 95% CIs around the point estimates. Therefore, the results have to be interpreted with caution. Third, because of the uneven distribution of displaced survivors in the community, we were unable to determine the precise threshold between 8 and 21 IDPs when the erosion of trust began to occur. The results suggest that the resettlement of a few scattered individuals in a community was not associated with changes in on local trust. The erosion of trust seemed to appear when larger numbers of people moved in. Fourth, we do not have information on the residential movements of people younger than 65 years, which might not be correlated with the movement of people aged 65 years or older. On the other hand, two-thirds of the population of the city of Iwanuma were aged 65 years or older before the disaster, and the age structure of IDPs was similar. Fifth, it is not clear how participants define people from their community and people from other communities. By 2013, when the question was asked, IDPs had spent approximately 2.5 years in their new environment. Thus, the respondents may have perceived the displaced population as either people from their own community or as outsiders. Hence, the 2 questions about trust might not have distinguished between particularized and generalized trust, which would also explain the similarity of the corresponding OR estimates. Also, perceived change in trust was measured based on 1 question instead of a multi-item scale, which hindered a more precise assessment of trust levels among the respondents. Sixth, the question of the generalizability of our results needs to be considered due to the relatively low response rate (59%) on the baseline survey. However, previous reports based on the JAGES study confirmed that the demographic profile of the participants is similar to the rest of the residents aged 65 years or more in Iwanuma City.^7,10^ Moreover, a 59% response rate is comparable with other studies on community-dwelling respondents.^29^

## Conclusions

In conclusion, while a well-designed group relocation is crucial for preserving social capital and ensuring better disaster resilience among displaced survivors, a higher concentration of IDPs might lead to the erosion of trust among the residents of the host community. To avoid the erosion of social cohesion, it may be crucial to provide programs for meaningful interactions between the old and new residents of the community.^26^ More research is needed to understand the short-term and long-term impact of different relocation practices on both displaced and nondisplaced survivors of disasters.

## Notes

Supplement. **eFigure.** Participant Flow Chart, Iwanuma, Japan, 2010-2013
