# Supplementary material for: Evaluation of Trust Within a Community After Survivor Relocation Following the Great East Japan Earthquake and Tsunami
Source: JAMA Netw Open. 2020 Nov 5;3(11):e2021166. doi: 10.1001/jamanetworkopen.2020.21166 (PMC7645695; doi:10.1001/jamanetworkopen.2020.21166)
Supplement: Supplement. — eFigure. Participant Flow Chart, Iwanuma, Japan, 2010-2013 [file jamanetwopen-e2021166-s001.pdf]

Supplemental Online Content

Gero K, Aida J, Kondo K, Kawachi, I. Evaluation of trust within a community after survivor relocation following the Great East Japan Earthquake and Tsunami. *JAMA Netw Open*. 2020;3(11):e2021166. doi:10.1001/jamanetworkopen.2020.21166

eFigure. Participant Flow Chart, Iwanuma, Japan, 2010-2013

This supplemental material has been provided by the authors to give readers additional information about their work.

© 2020 Gero K et al. *JAMA Network Open.*

eFigure. Participant flow chart, Iwanuma, Japan, 2010-2013

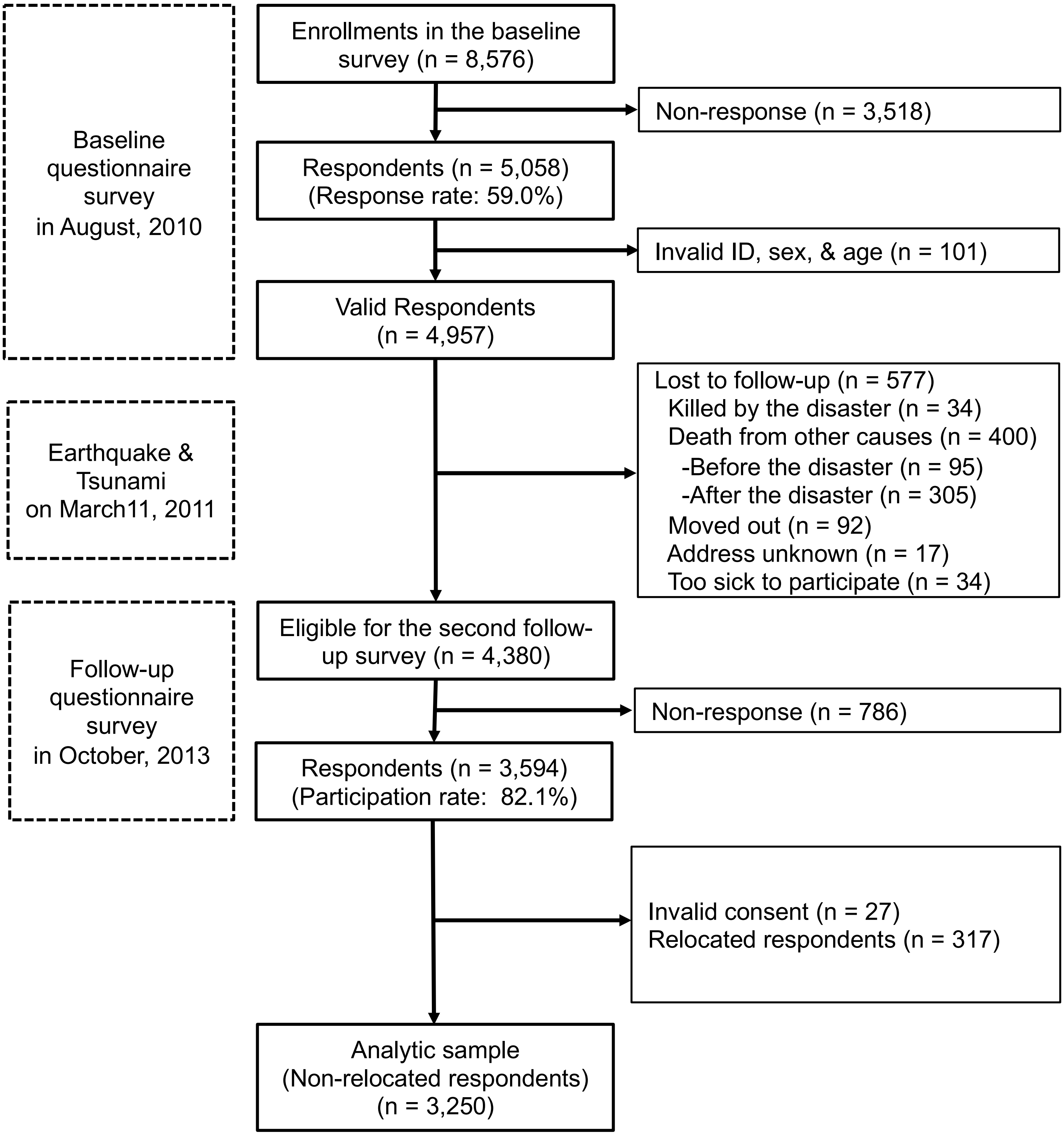

© 2020 Gero K et al. *JAMA Network Open.*
